# Carbon-Ion Beam Irradiation Alone or in Combination with Zoledronic acid Effectively Kills Osteosarcoma Cells

**DOI:** 10.3390/cancers12030698

**Published:** 2020-03-16

**Authors:** Eun Ho Kim, Mi-Sook Kim, Akihisa Takahashi, Masao Suzuki, Guillaume Vares, Akiko Uzawa, Akira Fujimori, Tatsuya Ohno, Sei Sai

**Affiliations:** 1 Department of Biochemistry, School of Medicine, Daegu Catholic University, Nam-gu, Daegu 42472, Korea; 2 Department of Radiation Oncology, Korea Institute of Radiological and Medical Sciences, Seoul 139-706, Korea; mskim@kirams.re.kr; 3 Gunma University Heavy Ion Medical Center, 3-39-22 Showa-machi, Maebashi 371-8511, Gunma, Japan; a-takahashi@gunma-u.ac.jp; 4 Department of Basic Medical Sciences for Radiation Damages, National Institute of Radiological Sciences, National Institutes for Quantum and Radiological Science and Technology, Chiba 263-8555, Japan; suzuki.masao@qst.go.jp (M.S.); uzawa.akiko@qst.go.jp (A.U.); fujimori.akira@qst.go.jp (A.F.); 5 Cell Signal Unit, Okinawa Institute of Science and Technology Graduate University (OIST), Onna-son 904-0495, Okinawa, Japan; guillaume.vares@oist.jp; 6 Department of Radiation Oncology, Gunma University Graduate School of Medicine, 3-39-22 Showa-machi, Maebashi 371-8511, Gunma, Japan; tohno@gunma-u.ac.jp

**Keywords:** zoledronic acid, osteosarcoma cells, bone cancer, carbon-ion, miR-29b

## Abstract

Osteosarcoma (OSA) is the most common malignant bone tumor in children and adolescents. The overall five-year survival rate for all bone cancers is below 70%; however, when the cancer has spread beyond the bone, it is about 15–30%. Herein, we evaluated the effects of carbon-ion beam irradiation alone or in combination with zoledronic acid (ZOL) on OSA cells. Carbon-ion beam irradiation in combination with ZOL significantly inhibited OSA cell proliferation by arresting cell cycle progression and initiating KHOS and U2OS cell apoptosis, compared to treatments with carbon-ion beam irradiation, X-ray irradiation, and ZOL alone. Moreover, we observed that this combination greatly inhibited OSA cell motility and invasion, accompanied by the suppression of the Pi3K/Akt and MAPK signaling pathways, which are related to cell proliferation and survival, compared to individual treatments with carbon-ion beam or X-ray irradiation, or ZOL. Furthermore, ZOL treatment upregulated microRNA (miR)-29b expression; the combination with a miR-29b mimic further decreased OSA cell viability via activation of the caspase 3 pathway. Thus, ZOL-mediated enhancement of carbon-ion beam radiosensitivity may occur via miR-29b upregulation; co-treatment with the miR-29b mimic further decreased OSA cell survival. These findings suggest that the carbon-ion beam irradiation in combination with ZOL has high potential to increase OSA cell death.

## 1. Introduction

Osteosarcoma (OSA) is the most frequent primary malignant bone tumor in children and adolescents [1,2]. Despite significant improvements in both diagnosis and treatment, overall survival is still unsatisfactory for advanced-stage OSA patients [3,4]. OSA is characterized by typical radioresistant tumors, and conventional radiation therapy is not effective for its treatment [5,6,7,8]. However, an increasing amount of evidence has demonstrated that high linear energy transfer (LET) carbon-ion radiation therapy is suitable for targeting many kinds of radioresistant tumors such as those associated with bone and soft tissue malignancies including OSA [9,10,11,12,13,14] because it primarily does not elicit cell cycle- and oxygen-dependent cell-killing effects; it also has a high potential to kill radiochemo-resistant cancer stem cells (CSCs) compared to low LET radiation [15,16,17,18,19]. However, although carbon ion beam radiotherapy treatment has yielded promising results, the prognosis of OSA still remains unsatisfactory; developing novel combinational therapeutic strategies to further improve overall survival is required.

Bisphosphonates comprise the most important class of osteoclast-mediated bone resorption inhibitors and are used extensively for treating skeletal diseases such as postmenopausal osteoporosis and tumor-induced osteolysis [20,21]. Zoledronic acid (ZOL), a third-generation nitrogen-containing bisphosphonate, is an inhibitor of osteoclast-mediated bone resorption that has demonstrated efficacy in treating bone metastases in cancer patients with breast, prostate, lung, and other solid tumors [22,23,24]. ZOL significantly enhances radiation-induced apoptosis and decreases cell viability, suggesting that it may exhibit radiosensitizing effects [25,26,27,28,29,30,31,32,33]. We have previously reported that ZOL enhanced γ-ray radiation-induced DNA damage and suppressed OSA cell migration and invasion [34,35]. Recently, we also found that it significantly induced autophagy via modulation of microRNA (miRNA) expression [36]. In addition, increasing evidence showed that some miRNAs are involved in regulating tumor progression and relapse [37]. miR-29b, a member of the miR-29 family, has been reported to be positively correlated with radiation-induced PTEN expression in lung cancer [38], and also promotes radiosensitivity via enhancing oxidative stress and inhibiting DNA damage repair in radioresistant cervical cancer [39]. Based on this finding, and in connection with above findings that miR-29b increases radiosensitivity [38,39], we hypothesize that a combination treatment strategy that includes ZOL and an miR-29b mimic may dramatically enhance carbon-ion beam radiosensitivity to achieve increased OSA cell death.

## 2. Results

### 2.1. Expression of MiR-29b and Its Role in OSA Cell Proliferation and Apoptosis

**Figure 1:**
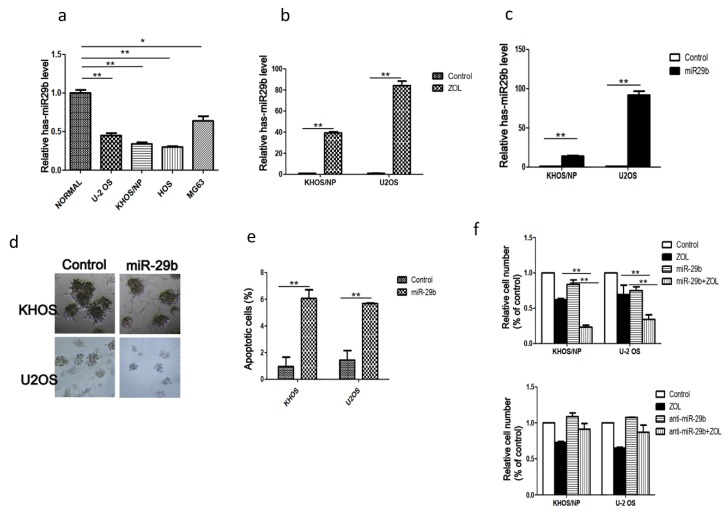
miR-29b expression and its involvement in osteosarcoma (OSA) cell viability after treatment of OSA cells with or without zoledronic acid (ZOL). (**a**) miR-29b expression levels were determined by qRT-PCR in normal and OSA cell lines. (**b**) miR-29b expression was upregulated in OSA cell lines by treatment with zoledronic acid (ZOL). (**c**) Increased miR 29b expression was confirmed 24 h after treatment with the miR-29b mimic. (**d**) Morphological changes of two OSA cell types after transfection with the miR-29b mimic were determined by 3D culture assay. (**e**) Increased apoptosis was seen after treatment with the miR-29b mimic in OSA cells compared to the control. (**f**) Proliferation of the two OSA cell lines were measured by Trypan blue assay after treatment with the miR-29b mimic, miR-29b inhibitor, and ZOL alone or the combination treatment. * *p* < 0.05, ** *p* < 0.001.

To determine whether miR-29b is involved in regulating the radiosensitization effects of ZOL, we examined miR-29b expression levels in several OSA cell lines (KHOS, U2OS, HOS, MG63) and also in 10 pairs of OSA tissues and matched adjacent histologically normal tissues by qRT-PCR. We found that miR-29b levels were significantly lower in the OSA cell lines and tumor tissues than in non-malignant tissues (Figure 1a,b). To determine whether miR-29b is involved in regulating ZOL-mediated OSA-cell radiosensitivity, we treated OSA cells with ZOL and the result indicates that ZOL treatment upregulated miR-29b expression (Figure 1b). Then, we transfected a miR-29b mimic into KHOS and U2OS cells and confirmed that this process significantly increased its expression level (Figure 1c). Subsequent assays revealed that the miR-29b mimic significantly inhibited cell growth and increased apoptosis in OSA cells (Figure 1d,e), and overexpression of miR-29 and ZOL treatment resulted in a significant decrease in proliferation in two OSA cells (Figure 1f).

### 2.2. Apoptosis Induction and Cell Cycle Aberration after Treatment with Carbon-Ion Beam Irradiation Alone or in Combination with ZOL in OSA Cells

**Figure 2:**
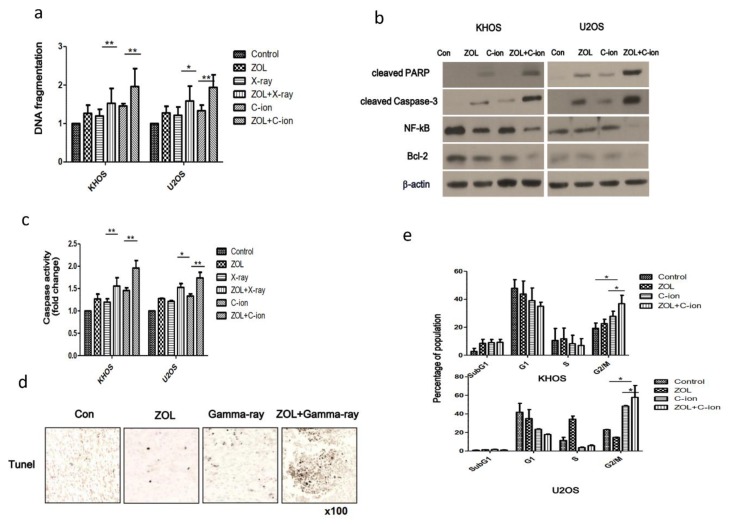
Apoptosis and cell cycle analyses after treatment with carbon-ion beam or X-ray or γ-ray irradiation alone or in combination with ZOL (**a**) DNA fragmentation assay was performed 48 h after the treatment of two OSA cell lines with carbon-ion beam (2 Gy) or X-ray (4 Gy) irradiation alone or in combination with ZOL (20 μM). (**b**) Western blotting for the quantification of apoptosis-related proteins after treatment with carbon-ion beam irradiation alone or in combination with ZOL. (**c**) Caspase 3 activity assay examined after treatment with carbon-ion beam and X-ray irradiation alone or in combination with ZOL. (**d**) TUNEL assays were performed using xenograft tumor tissues. Values represent the means of three experiments ± SD; * *p* < 0.05, ** *p* < 0.001. (**e**) Cell cycle analysis was performed after treatment with carbon-ion beam irradiation alone or in combination with ZOL by flow cytometry.

To confirm whether the ZOL combination treatment enhanced carbon-ion beam radiosensitivity, we examined apoptosis by using DNA fragmentation induction, caspase 3 activity assay, and apoptosis-related protein induction by western blot assay, following treatment of the cells with carbon-ion beam irradiation alone or in combination with ZOL (Figure 2a–c). The data showed that carbon-ion beam irradiation combined with ZOL significantly resulted in a relatively higher extent of DNA fragmentation, higher level of caspase activity, higher levels of cleaved caspase 3 and cleaved polyADP ribose polymerase (PARP), and lower B cell lymphoma-2 (Bcl-2) and NF-κB expression, compared to the individual treatments with carbon-ion beam irradiation or ZOL (*p* < 0.05). We also confirmed that the combination of γ-ray irradiation and ZOL increased the level of apoptosis in vivo by performing the TUNEL assay (Figure 2d). Furthermore, we performed cell cycle analysis and the data revealed that treatment with carbon-ion beam irradiation combined with ZOL increased the number of cells in the G2/M phase compared to the case for the treatment with carbon-ion beam irradiation or ZOL treatment alone, suggesting that combination treatment significantly attenuated cell cycle progression (Figure 2e). 

### 2.3. Involvement of PI3K–Akt and MAPK Signaling Pathways in OSA Cell Death after Carbon-Ion Beam Irradiation Alone or in Combination with ZOL 

**Figure 3:**
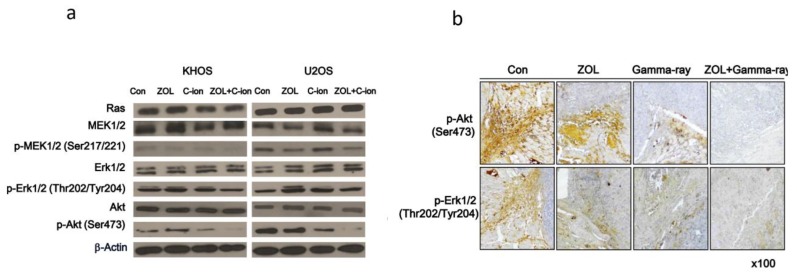
Phosphorylation of the PI3K-Akt and MAPK pathways after treatment of OSA cells with carbon-ion beam or γ-ray irradiation alone or in combination with ZOL. (**a**) Western blotting for the quantification of MAPK and Akt signaling-related proteins was performed after treatment of the OSA cells with carbon-ion beam irradiation alone or in combination with ZOL using the indicated antibodies. (**b**) p-AKT and p-ERK expression in xenograft tumors were examined by immunohistochemistry. Representative images are provided, as indicated.

To investigate the molecular mechanisms of ZOL carbon-ion beam radiosensitization, we investigated PI3K-Akt- and MAPK-signaling response after treatment with carbon-ion beam irradiation alone or in combination with ZOL in OSA cell lines. We found that carbon-ion beam irradiation combined with ZOL significantly decreased p- MAPK kinase (MEK)1/2, p- extracellular signal-related kinase (ERK)1/2, and p-Akt levels compared to treatment with carbon-ion beam irradiation alone (Figure 3a). In addition, γ-ray irradiation combined with ZOL significantly inhibited the expression of p-ERK1/2, and p-Akt in mouse xenografts tumors by immunohistochemical staining (Figure 3b).

### 2.4. Inhibition of OSA Cell Motility, Invasion, and Angiogenesis after Treatment with Carbon-Ion Beam Irradiation Alone or in Combination with ZOL

**Figure 4:**
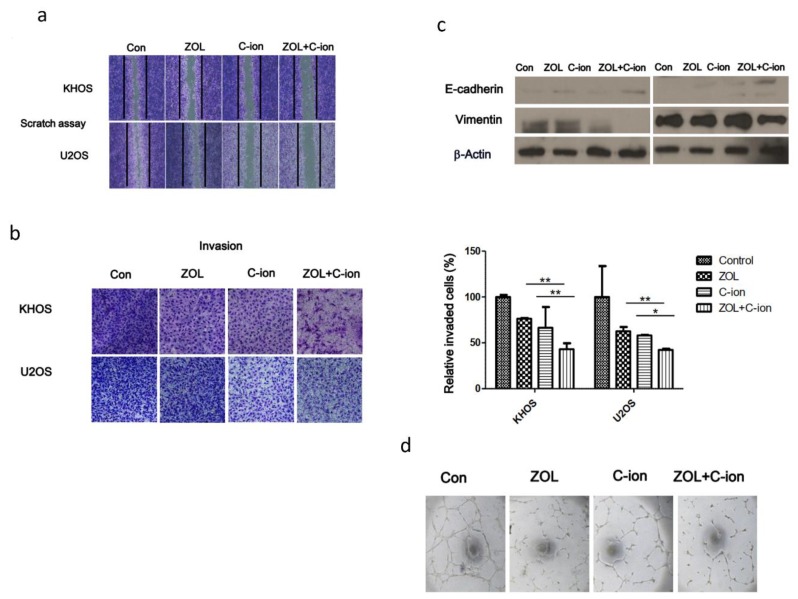
Cell invasion and migration analysis after treatment of OSA cells with carbon-ion beam irradiation alone or in combination with ZOL. (**a**) Wound-healing assay was performed by scraping the OSA cell layers with 200-μL pipette tips after treatment with carbon-ion beam irradiation alone or in combination with ZOL. The number of cells that migrated across the wound was counted after 24 h. Each sample was photographed, the distances between the migrating cell edges were quantified, and the percentage of cell migration was calculated. (**b**) OSA cell invasion and migration was examined by the transwell chamber assay 24 h after treatment of the cells with carbon-ion beam irradiation alone or in combination with ZOL. The number of invading tumor cells that penetrated through the Matrigel and gelatin were counted in five high-magnification microscopic fields. (**c**) Western blotting for the quantification of the epithelial to mesenchymal transition (EMT) markers E-cadherin and Vimentin performed after treatment of the cells with carbon-ion beam irradiation alone or in combination with ZOL using the indicated antibodies. (**d**) in vitro tube-formation assay after treatment of the cells with carbon-ion beam irradiation alone or in combination with ZOL using endothelial cells. Values represent the means of three experiments ± SD; *p < 0.05, **p < 0.001.

To determine the effects of treatment with carbon-ion beam irradiation alone or in combination with ZOL on OSA cell invasiveness and migration, wound-healing, transwell chamber, and matrigel-based in vitro endothelial tube-formation assays were performed. We found that carbon-ion beam irradiation combined with ZOL remarkably inhibited OSA cell migration and invasion, whereas treatment with carbon-ion beam irradiation and ZOL alone only slightly inhibited OSA cell migration and invasion (Figure 4a,b). Interestingly, western blotting and immunohistochemistry analysis showed that carbon-ion beam irradiation combined with ZOL upregulated the epithelial marker E-cadherin but downregulated the expression of the mesenchymal marker, vimentin, compared with controls (Figure 4c). A Matrigel-based tube formation assay using human tumor endothelial cells (2H11) + U2OS coculture system for detecting the angiogenesis which is critical for metastasis showed that TTF suppressed vascular tubule development (Figure 4d). Tube formation in the combined treatment group was decreased compared to that of the control or single treatment.

### 2.5. Effects of ZOL Alone or in Combination with Carbon-Ion Beam Irradiation on OSA Cell Proliferation 

**Figure 5:**
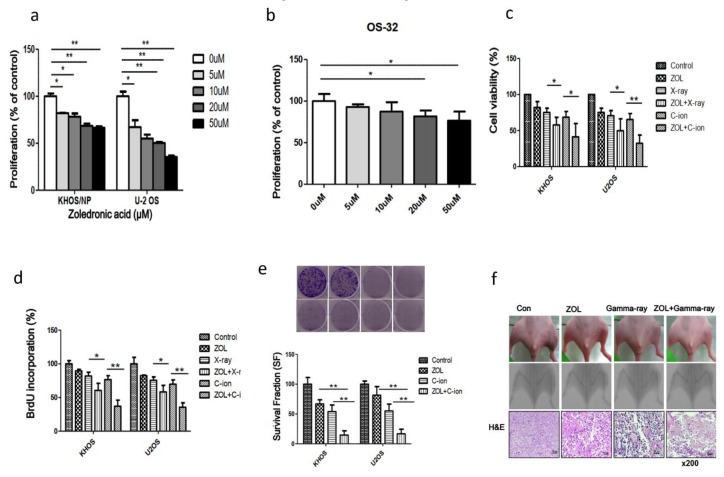
OSA cell proliferation, viability, and survival fraction after treatment with ZOL alone or in combination with carbon-ion beam or γ-ray irradiation: (**a**) proliferation of KHOS and U2OS cell lines and (**b**) an OSA-patient-derived cell line following treatment with different concentrations of ZOL for 72 h was measured by Trypan blue assay; (**c,d**) viability and proliferation of two OSA cell lines were measured by Trypan blue staining and BrdU incorporation assays after treatment with carbon-ion beam irradiation, X-ray, and ZOL alone or combination treatments. (**e**) Survival fraction of two OSA cell lines was measured by colony forming assay after treatment with carbon-ion beam irradiation alone or carbon-ion beam irradiation in combination with ZOL. (**f**) Morphological changes of KHOS orthotopic tumors after treatment with ZOL alone or in combination with γ-ray irradiation. KHOS cells were injected into the proximal tibia of four groups of nude mice (n = 3 each) to generate an orthotopic tumor model. The dimensions of the leg (including the tumor) were measured every 7 days by X-ray analysis. Representative radiographs of the limb of a mouse at 0 and 5 weeks after tumor inoculation are shown. Representative images of animal tumors at 5 weeks and a graph of tumor size against time are shown. Tumors were excised and processed for hematoxylin and eosin staining. Original magnification, 100×. For each assay, values represent the means of three experiments ± SD; * *p* < 0.05, ** *p* < 0.001.

KHOS and U2OS cells and cells from an OSA patient were treated for 48 h with various doses of ZOL, after which cell viability was analyzed by trypan blue staining assays (Figure 5a,b). We found that ZOL significantly inhibited the growth of cells treated with ≥ 5 µg/mL ZOL (*p* < 0.05), indicating a concentration-dependent sensitivity of the OSA cells to ZOL. Then, we examined the viability and proliferation of OSA cells after treatment with carbon-ion beam irradiation alone or carbon-ion beam irradiation in combination with 20 µM ZOL compared to that of X-ray treatment by trypan blue staining, and BrdU and clonogenic survival assays (Figure 5c–e). We found that the combination of carbon-ion beam irradiation and ZOL markedly decreased OSA cell viability and survival, compared to treatment with carbon-ion beam irradiation alone or treatment with X-ray irradiation and ZOL (Figure 5d,e).

We also examined the antitumor effect of ZOL in vivo by using an orthotopic mouse model, and found that ZOL treatment alone decreased tumor size and, in combination with γ-ray irradiation, it further reduced both tumor size and leg dimensions (Figure 5f).

### 2.6. Suppression of OSA Cell Proliferation and Induction of Apoptosis After Treatment with Carbon-Ion Beam Irradiation Alone or in Combination with ZOL and the MiR-29b Mimic 

**Figure 6:**
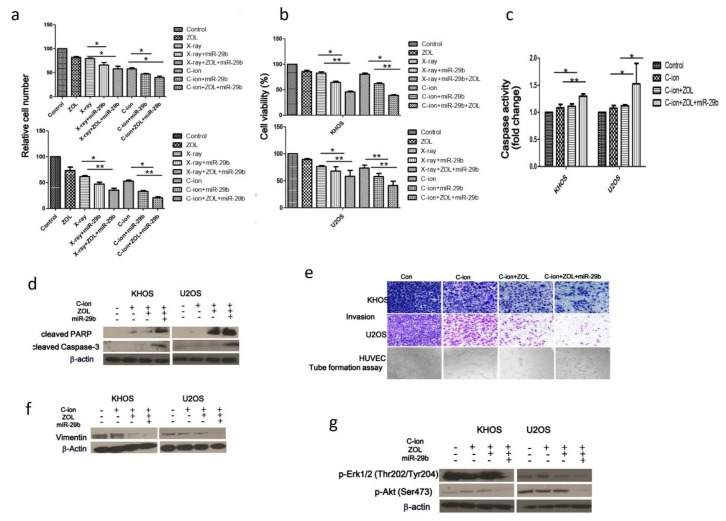
Effects of carbon-ion beam or X-ray irradiation alone or in combination with ZOL and miR-29b mimic on OSA cells. (**a,b**) Cell proliferation and viability of two OSA cell lines were measured by Trypan blue assay 72 h after treatment with carbon-ion beam irradiation alone or in combination with the miR-29b mimic and/or ZOL. (**c**) Caspase 3 activity assay examined after treatment with carbon-ion beam irradiation alone or in combination with ZOL. (**d**) Western blotting for the quantification of apoptosis-related proteins after treatment of the cells with carbon-ion beam irradiation alone or in combination with miR-29b mimic and/or ZOL. (**e**) OSA cell invasion and migration were examined by transwell chamber assay 24 h after treatment with carbon-ion beam irradiation alone or in combination with miR-29b mimic and/or ZOL. (**f**) Western blotting for the quantification of the epithelial to mesenchymal transition (EMT) markers Vimentin after treatment with carbon-ion beam irradiation alone or in combination with the miR-29b mimic and/or ZOL using the indicated antibodies. (**g**) Western blotting for the quantification of MAPK and Akt signaling-related proteins performed after treatment with carbon-ion beam irradiation alone or in combination with the miR-29b mimic and/or ZOL using the indicated antibodies.

To investigate whether treatment with the miR-29b mimic alone or in combination with ZOL increases radiosensitivity of OSA cells to carbon-ion beam irradiation, a cell viability assay and apoptosis analysis were performed. We found that carbon-ion beam irradiation combined with treatment with the miR-29b mimic significantly reduced cell viability, enhanced caspase-3/7 activity, and increased the cleaved caspase-3 and PARP levels; addition of ZOL further accentuated these effects (Figure 6a–d). Western blotting and transwell assays revealed that the miR-29b mimic decreased that of vimentin, and the triple combination treatment (carbon-ion beam + miR-29b mimic + ZOL) markedly enhanced these effects (Figure 6e,f). We also found that the triple combination treatment (carbon-ion beam + miR-29b mimic + ZOL) remarkably suppressed the MAPK- and PI3K-Akt-signaling pathways by decreasing the levels of p-ERK1/2 and p-Akt (Ser473) (Figure 6g).

## 3. Discussion

In the present study, we found that treatment with the combination of carbon-ion beam irradiation and ZOL, a third-generation bisphosphonate, notably decreased OSA cell viability and survival, with a greater extent of DNA fragmentation, higher caspase 3 activity, higher cleaved caspase 3 and cleaved PARP levels, and lower Bcl-2 and nuclear factor (NF)-κB expression levels than that caused by treatment with carbon-ion beam irradiation alone or X-ray irradiation combined with ZOL. In addition, treatment with carbon-ion beam irradiation combined with ZOL increased the number of cells in the G2/M phase, compared to the case for treatment with carbon-ion beam irradiation and ZOL alone, suggesting that the combination treatment significantly attenuated cell cycle progression. These findings are partially consistent with previous reports stating that ZOL augments the X-ray or γ-ray radiosensitivity of fibrosarcoma and nasopharyngeal carcinoma cells [22,23,24,25,26], indicating that ZOL not only enhances low LET photon beam radiosensitivity, but also high LET carbon-ion beam radiosensitivity in OSA cells. 

The PI3K/Akt and MAPK signaling pathways play an important role in cell survival and proliferation [40]. To clarify the molecular mechanisms of how ZOL enhances carbon-ion beam radiosensitivity, we investigated the involvement of the PI3K/Akt- and MAPK-signaling pathways; our data demonstrated that carbon-ion beam irradiation combined with ZOL significantly decreased the p-MEK1/2, p-ERK1/2, and p-Akt levels, compared to treatment with carbon-ion beam irradiation alone. These data suggest that ZOL acts as an inhibitor of both the PI3K/Akt- and MAPK-signaling pathways, thereby enhancing the effects of carbon-ion beam irradiation on OSA cells. These results support the findings of recent studies that the combination treatment with ZOL increased cytotoxic effects via suppression of the Pi3K/Akt and MAPK signaling pathways [24,26,41,42]. 

The prognosis of OSA patients with metastasis is very poor, with the overall 5-year survival rate being only 10–30% [1,2,3]. Thus, it is very important to control tumor invasion and metastasis. In this study, the effects of treatment with carbon-ion beam irradiation alone or in combination with ZOL on OSA cell invasiveness and migration were investigated. We found that carbon-ion beam irradiation combined with ZOL remarkably inhibited OSA cell migration and invasion, whereas individual treatments with carbon-ion beam irradiation and ZOL only slightly inhibited OSA cell migration and invasion. Interestingly, western blotting and immunohistochemistry analysis showed that carbon-ion beam irradiation combined with ZOL upregulated the expression of EMT markers such as E-cadherin but downregulated vimentin. Our findings are consistent with those of previous reports, which stated that ZOL exhibits antitumor and antimetastatic activities [25,32,43,44]. 

Interestingly, in this study, we found that treatment with ZOL effectively inhibited the Pi3K/Akt and MAPK signaling pathways, and significantly increased miR-29b expression in OSA cells. This is partially consistent with previous studies that ZOL suppresses Erk1/2 and PI3K/Akt pathway in cervical cancer [45] and upregulates PTEN via the downregulation of Akt in breast cancer [46]. Since PTEN is positively correlated with the upregulation of miR-29b in lung cancer and tongue squamous cancer [38,40], we speculate that ZOL-enhanced PTEN levels might subsequently result in the upregulation of miR-29b. Based on the recent reports that miR-29b exerts anticancer effects and augments radiosensitivity in glioblastoma and cervical cancer cells [47,48], we hypothesized that modulation of miR-29b expression may further increase ZOL-mediated radiosensitization; hence, we investigated whether treatment with the miR-29b mimic combined with ZOL further increased carbon-ion beam radiosensitivity of OSA cells. As we expected, carbon-ion beam irradiation combined with miR-29b mimic treatment significantly inhibited cell viability, enhanced caspase-3/7 activity, and increased the levels of cleaved caspase-3 and PARP; the addition of ZOL further enhanced these effects. Western blot and transwell assays revealed that the miR-29b mimic increased the expression of E-cadherin but decreased that of vimentin, and the triple combination treatment (carbon-ion beam + miR-29b mimic + ZOL) predominantly enhanced these effects. We also found that the triple combination treatment (carbon-ion beam + miR-29b mimic + ZOL) remarkably suppressed the mitogen-activated protein kinase (MAPK)- and PI3K/Akt-signaling pathways by decreasing the levels of p-ERK1/2 and p-Akt (Ser473). 

In summary, the third-generation bisphosphonate ZOL effectively enhanced the radiosensitivity of OSA cells to carbon-ion beam irradiation; additional treatment with an miR-29b mimic further increased the OSA cell-killing effects, implicating that a combination treatment not only with traditional anticancer agents but also with molecular targeting agents such as the tumor suppressor miR-29b has a high potential to enhance the cancer cell-killing effects of carbon-ion beam radiotherapy.

## 4. Materials and Methods 

### 4.1. Cell Culture and Tissue Samples

Two OSA cell lines were selected for this study. U2OS and KHOS/NP OSA cells were obtained from the American Type Culture Collection (Rockville, MD, USA) and maintained in α minimum essential medium (Gibco; Life Technologies, Carlsbad, CA, USA) containing 10% (v/v) fetal bovine serum (Gibco; Life Technologies) and 1% (v/v) penicillin-streptomycin (Gibco; Life Technologies). OSA tissue was obtained with informed consent from a patient who underwent surgery at the Korea Institute of Radiological and Medical Sciences (Institutional Review Board approval number: K-1603-001-001), and a primary cell culture was established from this tissue. Briefly, the tissue was minced into a slurry with blades, washed with phosphate-buffered saline (PBS), and centrifuged for 3 min at 1000 rpm. The supernatant was then discarded, and the pellet was resuspended in serum-free Dulbecco’s modified Eagle medium (WelGene, Daegu, Korea) containing from 0.05% to 0.1% (w/v) type-I collagenase (Gibco; Life Technologies) in order to disaggregate the cells. After 2 h, the cells were washed thoroughly with PBS and maintained in Dulbecco’s modified Eagle medium containing 20% (v/v) fetal bovine serum.

### 4.2. Reagents

Anti-NF-κB, anti- Bcl-2, anti-phospho-Raf-1, anti-Akt, anti-vimentin, and anti-β-actin antibodies were purchased from Santa Cruz Biotechnology (Dallas, TX, USA). Anti- cleaved PARP, anti-caspase-3, anti-Ras, anti-MEK1/2, anti-p-MEK1/2, anti-ERK1/2, anti-p-ERK1/2, anti-p-Akt(Ser473), and anti-E-cadherin antibodies were purchased from Cell Signaling Technology (Danvers, MA, USA), and anti-γ-H2A histone family member X antibodies were obtained from Millipore (Billerica, MA, USA). ZOL was purchased from Sigma–Aldrich (St. Louis, MO, USA). For in vitro experiments, ZOL was dissolved in PBS to prepare a 2 mM stock solution and stored at −20 °C.

### 4.3. Irradiation 

Cells were plated in 60-mm plastic dishes and incubated at 37 °C under a humidified 5% CO_2_ atmosphere. For in vitro experiments, cells at 70–80% confluence were irradiated with X-rays (Titan-320, GE Co., USA) at a dose rate of 2.45 Gy/min. Cells were irradiated with 290 MeV/n, 6 cm-Spread Out Bragg Peak (SOBP) carbon-ion beams with a dose-averaged LET of 50 keV/μm at a dose rate of 2.0–5.0 Gy/min at the Heavy Ion Medical Accelerator in Chiba, Japan (HIMAC). For in vivo experiments, mice were irradiated using a ^60^Co γ-ray source (Theratron 780, Atomic Energy of Canada, Chalk River, Ontario, Canada) with a 0.5 cm diameter bolus of tissue equivalent material to allow for dose buildup. A lead barrier was used to shield normal tissues where possible.

### 4.4. Cell-Viability Assay

Cells were seeded at a density of 5000 cells/well in a 96-well plate and incubated for 24 h according to the indicated experimental conditions. For the quantification of cell viability, an equal volume of culture medium containing EZ-Cytox reagent (EZ3000; Daeillab Service, Seoul, Korea) was added to the cells, and the mixture was incubated for 4 h. The cell viability was then assessed by measuring the absorbance of the samples using a Multiskan EX instrument (Thermo Fisher Scientific, Waltham, MA, USA) at 450 nm.

### 4.5. 5-Bromo-2′-Deoxyuridine (BrdU)-Labeling Assay

BrdU-labeling assays were performed in 96-well plates using the BrdU cell proliferation assay kit (Cell Signaling Technology, Danvers, MA, USA). After ZOL treatment, 10 μM BrdU was added to each well, and the cells were incubated for 12 h at 37 °C. BrdU signaling was determined using a Multiscan FC ELISA reader (Thermo Fisher Scientific, Danvers, MA, USA) at 450 nm.

### 4.6. Colony Formation Assay 

Cells were treated with ZOL for 48 h and then incubated for 7 to 9 days, and the resulting colonies were stained with 0.4% crystal violet (Sigma–Aldrich) and counted. The plating efficiency represents the percentage of seeded cells that grew into colonies under the specific culture conditions for a given cell line. The survival fraction was calculated as follows: survival fraction = colonies counted/(cells seeded × plating efficiency/100).

### 4.7. Orthotopic Animal Model and Histological Analysis 

Twelve 4-week-old female Balb/c nude mice (average weight: 12.1 g; range: 11.3–13.1 g) were obtained from ORIENT Bio (Seoul, Korea) and quarantined for 1 week prior to experimentation. KHOS/NP orthotopic tumors were established as previously described [37]. Briefly, mice were anesthetized by intraperitoneal injection of a mixture of zoletil (Virbac, Carros, France) and roumpun (Bayer Korea, Seoul, Korea). The left tibia was wiped with 70% (v/v) ethanol, and an 18-gauge needle was inserted through the tibial plateau with the knee flexed, followed by the injection of 1 × 10^5^ KHOS/NP cells resuspended in 10 μL of PBS into the marrow space of the proximal tibia using a 26-gauge needle coupled to a Hamilton syringe. Two weeks after the tumor cell inoculation, mice were randomly assigned into four groups (n = 3 each): control (untreated), ZOL treatment alone, ionizing radiation (IR) treatment alone, and combined ZOL and IR treatment (ZOL+IR). ZOL was administered intraperitoneally twice weekly at a dose of 0.1 mg/kg in 100 μL of PBS 2 weeks after inoculation and before and after irradiation, and IR was administered as a single dose of 8 Gy (γ-ray using a Co-60 source; 1.99 min/Gy) 2 weeks after inoculation. The animals were euthanized 6 weeks after tumor cell inoculation by CO_2_ asphyxiation. Tumor volumes were determined according to the formula (L × l2)/2 by measuring the tumor length (L) and width (l) with a pair of Vernier calipers after euthanasia. All experimental protocols were approved by the Institutional Animal Care and Use Committee of the Korea Institute of Radiological and Medical Sciences. Histological analysis was performed using hematoxylin and eosin-stained paraffin sections.

### 4.8. Caspase Activity Assay

Caspase activities were measured using caspase 3 activity assay kits (ab39401, Abcam, Cambridge, MA, USA) according to the manufacturer’s recommendations. Data were collected using a Multiskan EX at 405 nm.

### 4.9. Detection of Apoptotic Cells by Annexin V Staining

ZOL was added to the cells, which were then incubated for a further 48 h. The cells were washed with ice-cold PBS, trypsinized, and resuspended in 1× binding buffer [10 mM HEPES/NaOH (pH 7.4), 140 mM NaCl, and 2.5 mM CaCl_2_] at a density of 1 × 10^6^ cells/mL. Aliquots (100 μL) of the cell suspension were mixed with 5 μL of annexin V fluorescein isothiocyanate (BD Pharmingen, San Diego, CA, USA) and 10 μL of propidium iodide (PI) stock solution (50 μg/mL in PBS) by gentle vortexing, followed by a 15-min incubation at room temperature in the dark. Buffer (400 µL; 1×) was added to each sample, and the samples were analyzed on a FACScan flow cytometer (Becton Dickinson, Franklin Lakes, NJ, USA). A minimum of 10,000 cells were counted for each sample, and data analysis was performed using CellQuest software (BD Biosciences, San Jose, CA, USA). 

### 4.10. Western Blotting Analysis

ZOL was added to the OSA cells, which were then incubated for 24 h or 48 h. The cells were then lysed with radioimmunoprecipitation assay buffer, and proteins were separated by sodium dodecyl sulfate–polyacrylamide gel electrophoresis and transferred onto nitrocellulose membranes. The membranes were blocked with 1% (v/v) nonfat dried milk in Tris-buffered saline with 0.05% Tween20 and incubated with the required antibodies. Primary antibodies were used at a 1:1000 dilution, and secondary antibodies were used at a 1:5000 dilution. Immunoreactive protein bands were visualized by enhanced chemiluminescence (Amersham Biosciences, Little Chalfont, UK) and scanned.

### 4.11. Flow Cytometry

Cells were cultured, harvested at the indicated times, stained with PI (1 μg/mL; Sigma–Aldrich) according to manufacturer protocol, and analyzed using a FACScan flow cytometer (Becton Dickinson). A minimum of 10000 cells were counted for each sample, and data analysis was performed using CellQuest software (BD Biosciences).

### 4.12. TUNEL Assays

Tumors were collected and fixed with 10% neutral-buffered formalin. Deparaffinized sections were incubated with 20 μg/mL protease K for 15 min at room temperature, washed with PBS and incubated with terminal deoxynucleotidyl transferase-mediated dUTP nick end labeling (TUNEL) reaction mixture (Millipore, Burlington, MA, USA) for 1 h at 37 °C in a humidified chamber.

### 4.13. Wound-Healing (Scratch) Assay 

Human OSA cells were seeded onto 6-well plates (Corning, Corning, NY, USA) at a density of 2.5 × 10^4^ cells/well with 3 mL of medium. After pre-incubation with ZOL for 24 h, cells were irradiated, and on day 2, the monolayers were mechanically disrupted with a sterile 200-μL pipette tip. The assay was performed in duplicate, and wells were photographed every 48 h prior to staining with 0.2% (w/v) crystal violet. Cell migration was monitored using an Eclipse Ti microscope with a DS-Fi1 camera (Nikon, Tokyo, Japan), and migrated cells were counted using the ImageJ software (National Institutes of Health, Bethesda, MD, USA). 

### 4.14. Transwell Chamber Assay 

The invasive ability of OSA cells was measured using transwell chambers (Millipore) according to the manufacturer’s protocol. Briefly, cells were seeded onto the membrane of the upper chamber of the transwell at a concentration of 4 × 10^5^ cells/mL in 150 μL of the medium and left untreated or treated with the indicated doses of ZOL, IR, or ZOL+IR for 24 h. The medium in the upper chamber was serum free, whereas that in the lower chamber contained 10% (v/v) fetal bovine serum as a chemoattractant. Cells that passed through the Matrigel/gelatin-coated membrane were stained with cell stain solution containing crystal violet supplied in the transwell chamber assay (Chemicon; Millipore) and photographed after 24 h of incubation.

### 4.15. Matrigel-Based in Vitro Endothelial Tube-Formation Assay

Endothelial-cell tube formation was assessed using Matrigel-coated chamber slides, as previously described [38]. The results of each assay were photographed (Eclipse Ti microscope with a DS-Fi1 camera; Nikon) at 40× magnification.

### 4.16. miRNA and Transient Transfection

The miR-29b mimics, control mimics, miR-29b inhibitors, and control inhibitors were all purchased from Bioneer (Daejeon, Korea). Cells were transiently transfected with 60 nM control or miR-29b mimics, or with 120 nM control or miR-29b inhibitors, using X-treme GENE siRNA Transfection Reagent (Roche, Indianapolis, IN, USA).

### 4.17. Statistical Analysis

Statistical significance was determined using Student’s t test, and differences were considered significant at *p* < 0.05 or *p* < 0.001.
